# BAFF Promotes Th17 Cells and Aggravates Experimental Autoimmune Encephalomyelitis

**DOI:** 10.1371/journal.pone.0023629

**Published:** 2011-08-29

**Authors:** Xiaohui Zhou, Zanxian Xia, Qin Lan, Julie Wang, Wenru Su, Yuan-Ping Han, Huimin Fan, Zhongmin Liu, William Stohl, Song Guo Zheng

**Affiliations:** 1 Division of Rheumatology, Department of Medicine, University of Southern California Keck School of Medicine, Los Angeles, California, United States of America; 2 Shanghai East Hospital, Tongji University School of Medicine, Shanghai, China; 3 State Key Laboratory of Ophthalmology, Zhongshan Ophthalmic Center, Sun Yat-sen University, Guanzhou, China; 4 Department of Surgery, University of Southern California Keck School of Medicine, Los Angeles, California, United States of America; Universität Würzburg, Germany

## Abstract

**Background:**

BAFF, in addition to promoting B cell survival and differentiation, may affect T cells. The objective of this study was to determine the effect of BAFF on Th17 cell generation and its ramifications for the Th17 cell-driven disease, EAE.

**Methodology/Principal Findings:**

Th17 cells were increased in BAFF-Tg B6 (B6.BTg) mice and decreased in B6.*Baff^−/−^* mice. Th17 cells in B6.*Baff^−/−^* mice bearing a BAFF Tg (B6.*Baff^−/−^*.BTg mice) were identical to those in B6.BTg mice, indicating that membrane BAFF is dispensable for Th17 cell generation as long as soluble BAFF is plentiful. In T + non-T cell criss-cross co-cultures, Th17 cell generation was greatest in cultures containing B6.BTg T cells and lowest in cultures containing B6.*Baff^−/−^* T cells, regardless of the source of non-T cells. In cultures containing only T cells, Th17 cell generation followed an identical pattern. CD4^+^ cell expression of CD126 (IL-6R α chain) was increased in B6.BTg mice and decreased in B6.*Baff^−/−^* mice, and activation of STAT3 following stimulation with IL-6 + TGF-β was also greatest in B6.BTg cells and lowest in B6.*Baff^−/−^* cells. EAE was clinically and pathologically most severe in B6.BTg mice and least severe in B6.*Baff^−/−^* mice and correlated with MOG_35–55_ peptide-induced Th17 cell responses.

**Conclusions/Significance:**

Collectively, these findings document a contribution of BAFF to pathogenic Th17 cell responses and suggest that BAFF antagonism may be efficacious in Th17 cell-driven diseases.

## Introduction

B cell activating factor of the TNF family (BAFF, also known as BLyS) is a 285-amino acid type-II transmembrane protein member of the TNF ligand superfamily [1], [2] expressed by myeloid-lineage cells [1]–[5], BM-derived radiation-resistant stromal cells [6], and, to some degree, T cells [2], [7]. Cleavage of surface BAFF by a furin protease results in release of a soluble, biologically active 17-kDa molecule [2], [4] which binds to three receptors, BCMA, TACI, and BR3 (also known as BAFFR) on the surface of B cells [8]–[11]. BAFF is known best as a B cell survival factor [12]–[14], and it also plays important roles in B cell differentiation [15] and in Ig class switching and Ig production [16], [17]. In mice, BAFF deficiency leads to marked reductions in B cells beyond the T1 stage and marked reductions in baseline serum Ig levels and Ag-specific Ig responses [18], [19]. Conversely, exogenous BAFF enhances Ag-specific antibody responses [13], and repeated administration of BAFF leads to B cell expansion and polyclonal hypergammaglobulinemia [1].

Over-expression of BAFF is associated with autoimmunity in both mice and humans. Constitutive over-expression of BAFF in BAFF-Tg mice otherwise not autoimmune-prone leads to systemic lupus erythematosus (SLE)-like features [20]–[22], and treatment of SLE-prone mice with a BAFF antagonist ameliorates disease [22]–[24]. Moreover, many BAFF-Tg mice that do not succumb to SLE nephritis develop a Sjögren's syndrome-like phenotype [25], demonstrating that SLE is not the only autoimmune consequence of BAFF over-expression. Furthermore, BAFF over-expression is a feature of collagen-induced arthritis (CIA) in mice [26], with BAFF antagonism having both preventive and therapeutic effects [18], [27].

In humans, circulating BAFF levels are elevated in as many as 50% of SLE patients [28]–[30], and these levels correlate with clinical disease activity [31]. In addition, circulating BAFF levels are increased in substantial fractions of patients with a variety of rheumatic disorders, including rheumatoid arthritis [28], [29], Sjögren's syndrome [25], [32], and progressive systemic sclerosis [33].

In light of the central importance of T cells in multiple autoimmune diseases, the ability of BAFF to promote clinical disease (rather than just serological autoimmunity) suggests that BAFF affects not only B cells but T cells as well. Indeed, BAFF co-stimulates *in vitro* T cell proliferation [34], [35], and BAFF over-expression leads to skewing of *in vivo* inflammatory responses toward a Th1 cell profile and away from a Th2 cell profile [36]. Of note, complete B cell deficiency does not alter this Th1/Th2 polarizing effect of BAFF over-expression, demonstrating a potent BAFF-driven effect on T cells that is independent of B cells and may reflect, at least in part, a direct effect of BAFF on T cells themselves.

With the increasing appreciation for the importance of Th17 cells in autoimmune diseases, including EAE [37], the elucidation of the role for BAFF in Th17 cell responses becomes urgent. With that in mind, we investigated the effects of systemic BAFF over-expression or BAFF deficiency on Th17 cell generation and the attendant consequences for EAE.

## Materials and Methods

### General

All reported studies were approved by the University of Southern California Institutional Animal Care and Use Committee.

### Mice

C57BL/6 (B6) WT, B6.*Baff^−/−^*, and B6.BTg mice [19], [20], [38] were maintained in a single specific pathogen-free room. B6.*Baff^−/−^* mice bearing a BAFF Tg (B6.*Baff^−/−^*.BTg mice) were generated by intercrossing B6.*Baff^−/−^* and B6.BTg mice. The BAFF Tg in these mice is under the control of a liver-specific promoter containing the human ApoE enhancer and α-anti-trypsin [20], so B6.*Baff^−/−^*.BTg mice express large quantities of soluble BAFF but do not express membrane BAFF on their myeloid-lineage cells.

### Surface and intracellular staining

Spleen, LN, or peripheral blood cell populations were stained for surface or intracellular markers [39] with combinations of fluorochrome-conjugated mAb specific for CD126, CCR6, IFNγ (BD Biosciences Pharmingen, San Diego, CA), CD4, CD19, CD8a, IL-17A (BioLegend, San Diego, CA), RORγt, CD62L, CD44, CD3, or CD69 (eBioscience, San Diego, CA). In the case of intracellular IL-17 and IFNγ, isolated T cells were stimulated with PMA (0.25 µg/ml) + ionomycin (0.25 µg/ml) for 5 hr in the presence of brefeldin A (5 µg/ml; all from Calbiochem, La Jolla, CA) for the final 4 hr prior to processing for intracellular staining.

### Isolation of T cells and naive CD4^+^ cells

T cells were purified by negative selection by labeling nylon wool non-adherent spleen or LN cells with the combination of PE-conjugated anti-CD11b mAb (BioLegend) + PE-conjugated anti-B220 mAb (BD Biosciences Pharmingen), incubating the labeled cells with anti-PE magnetic beads, and trapping the labeled cells in a MACS magnetic bead separation column (Miltenyi Biotec, Auburn, CA). Naive CD4^+^ cells (CD4^+^CD25^−^CD44^lo^CD62L^+^) were purified by negative selection from nylon wool non-adherent LN cells as above, except that the cells were labeled with the combination of PE-conjugated anti-CD8 mAb + PE-conjugated anti-CD25 mAb + anti-CD11b mAb + PE-conjugated anti-B220 mAb + PE-conjugated anti-CD44 mAb. Purity of recovered cells was routinely >97%.

### *In vitro* induction of Th17 cells

Naive CD4^+^ cells were cultured for 3–4 days in the presence of irradiated non-T cells as APC (1∶1) with soluble anti-CD3 mAb + anti-CD28 mAb (1 µg/ml each), IL-6 (10 ng/ml), TGF-β (2 ng/ml), and anti-IL-4 and anti-IFNγ antibodies (10 µg/ml each) (R&D Systems, Minneapolis, MN) [39]. In some experiments, naive CD4^+^ cells were cultured as above, substituting plate-bound anti-CD3 (2 µg/ml) + soluble anti-CD28 (1 µg/ml) for irradiated non-T cells + soluble anti-CD3/CD28. Where indicated, exogenous BAFF (100 ng/ml) (R&D Systems) was added.

### Immunoblot analysis

Spleen T cells were cultured for 0–30 minutes with IL-6 (10 ng/ml, R&D Systems) or IL-6 + TGF-β (2 ng/ml, R&D Systems) and lysed by addition of M-PER Mammalian Protein Extraction Reagent (Thermo Scientific, Waltham, MA) in the presence of protease and phosphatase inhibitor cocktails (Sigma-Aldrich, St. Louis, MO) for 10 min on ice. After loading the SDS-PAGE loading buffer, the samples were heated at 95°C for 3 min and then subjected to SDS-PAGE and immunoblotting with the primary antibodies against phospho-Stat3, Stat3, phospho-Smad3 (Cell Signaling Technology, Danvers, MA), or GAPDH (Millipore, Billerica, MA). Signals were developed with SuperSignal West Femto Maximum Sensititivity Substrate (Thermo Scientific).

### Induction of EAE

On days 0 and 7, mice were injected s.c. with 100 µl of an emulsion of MOG_35–55_ peptide (1 mg/ml; CPC Scientific, San Jose, CA) in CFA in each of three points in the hind flank. Pertussis toxin (250 ng, List Biological Laboratories, Campbell, CA) was injected i.p. on days 0, 2, 7, and 9. Clinical signs were scored on a 0–5 scale as previously described [40].

### Ag-specific production of IL-17

Spleen or draining LN cells were cultured with MOG_35–55_ peptide (500 ng/ml) for 3 days. The culture supernatants were assayed for IL-17A by ELISA (eBioscience) according to the manufacturer's instructions, and the recovered CD4^+^ cells were immunostained for intracellular IL-17A as above.

### Histology and immunohistology

Paraffin sections of brain and spinal cord were stained with H&E or were immunostained with rabbit IgG anti-IL-17 antibodies (Santa Cruz Biotechnology, Santa Cruz, CA) followed by the universal immunoperoxidase ABC kit (Vector Laboratories, Burlingame, CA).

### Statistical analysis

All statistics were determined using software from GraphPad Software (La Jolla, CA). Parametric testing between two groups was performed by the unpaired t test. Parametric testing among three or more groups was performed by one-way ANOVA. For EAE scoring (which is ordinal rather than interval), non-parametric testing was performed by the Mann-Whitney rank sum test between two groups and by Kruskal-Wallis one-way ANOVA on ranks among three or more groups. P≤0.05 was considered to be significant.

## Results

### BAFF promotes T cell activation and expansion

**Figure 1:**
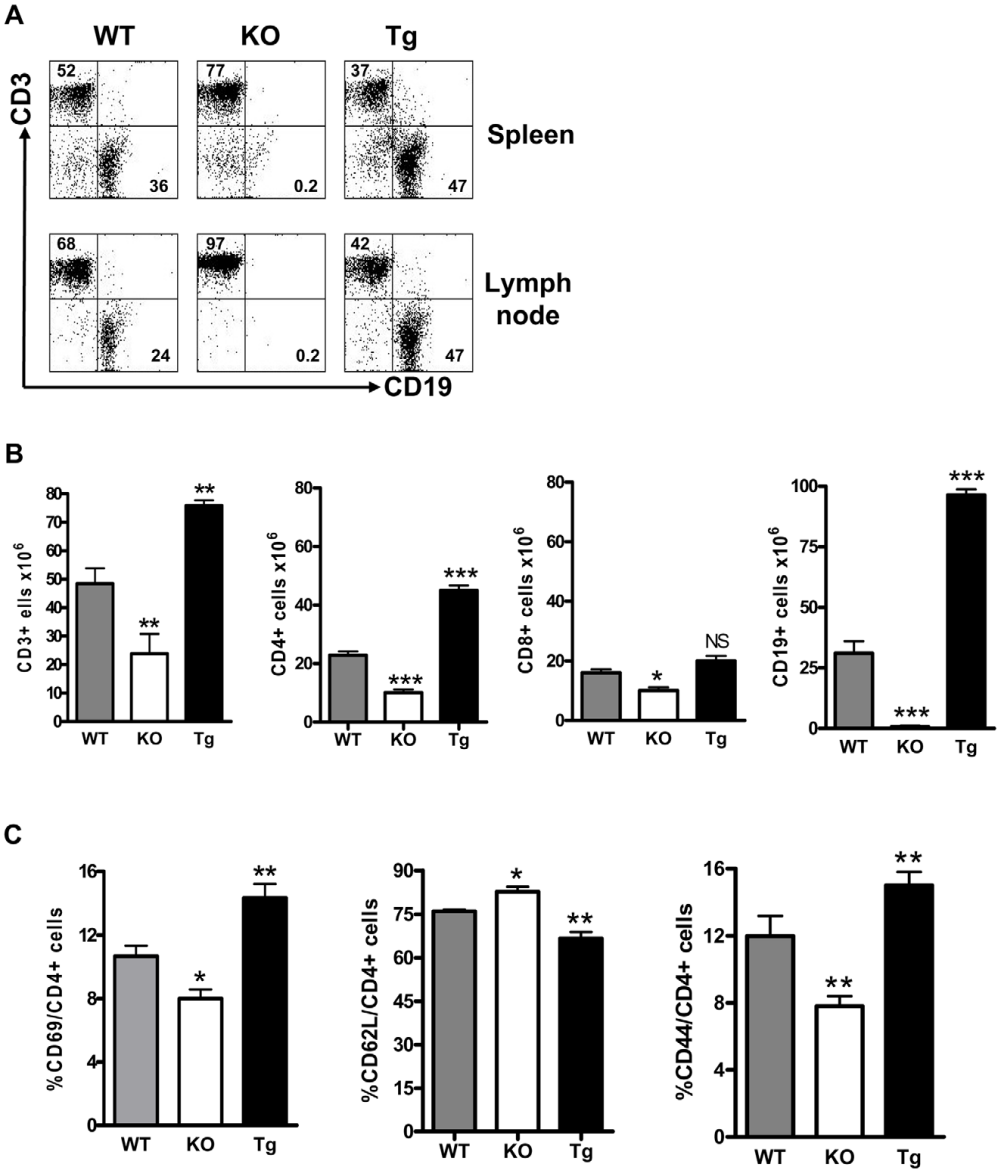
Decrease in both B cells and T cells in B6.*Baff^−/−^* mice and increase in both B cells and T cells in B6.BTg mice. Panel A: Spleen (top) or LN (bottom) cells from representative B6 WT (WT), B6.*Baff^−/−^* (KO), and B6.BTg (Tg) mice were stained for surface CD3 and surface CD19 expression. The values adjacent to the boxes in each tracing indicate the percentages of CD3^+^ or CD19^+^ cells in that sample. Panel B: Results (mean ± SEM) are presented for total spleen CD3^+^, CD4^+^, CD8^+^, and CD19^+^ cells for the 6 mice in each cohort. Panel C: Results (mean ± SEM) are presented for LN CD4^+^CD69^+^, CD4^+^CD44^hi^, and CD4^+^CD62L^lo^ cells for the 6 mice in each cohort. *, p≤0.05; **, p≤0.01; ***, p≤0.001 in comparison to WT values.

In agreement with the observations of others [19], [20], the percentages and total numbers of B (CD19^+^) cells in the spleens and LN of B6.*Baff^−/−^* and B6.BTg mice were markedly lower and substantially greater, respectively, than those in the spleens and LN of B6 WT mice (Figure 1A–B). Moreover, total numbers of T (CD3^+^) cells in B6.*Baff^−/−^* and B6.BTg mice also were lower and greater, respectively, than in B6 WT mice, with CD4^+^ cells being more affected than CD8^+^ cells by changes in BAFF. In addition, CD4^+^ cells from B6.*Baff^−/−^* mice displayed a less activated phenotype (fewer CD69^+^ and CD44^+^ cells and more CD62L^+^ cells) than did CD4^+^ cells from B6 WT mice, whereas CD4^+^ cells from B6.BTg mice displayed a more activated phenotype (more CD69^+^ and CD44^+^ cells and fewer CD62L^+^ cells) than did CD4^+^ cells from B6 WT mice (Figure 1C).

### BAFF-driven generation of Th17 cells

**Figure 2:**
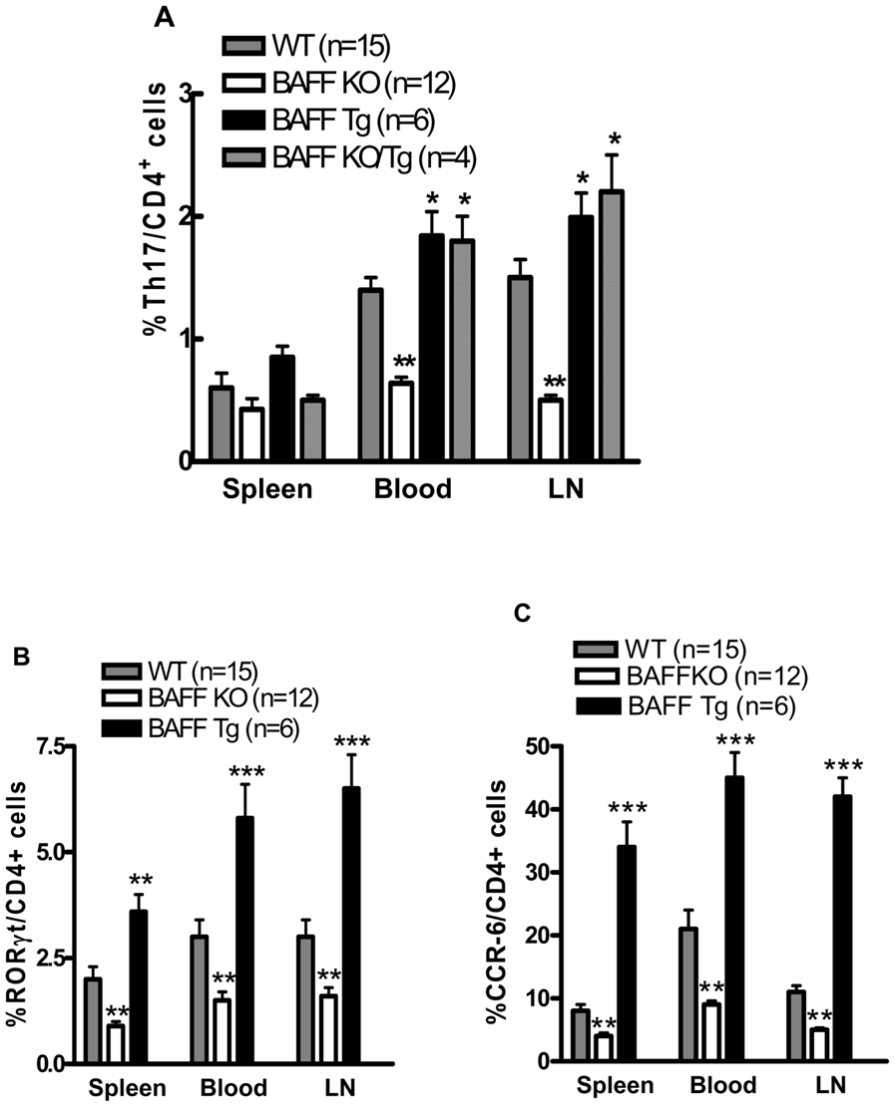
Promotion of Th17 cells by BAFF. T cells from spleen, blood, or LN of the indicated mice were cultured for 5 hr with PMA + ionomycin. Brefeldin A was added for the final 4 hr of culture to block secretion of cytokines and permit their accumulation intracellularly. Harvested cells were stained for surface CD4 and intracellular IL-17 (panel A), intracellular RORγt (panel B), or surface CCR-6 (panel C). *, p≤0.05; **, p≤0.01; ***, p≤0.001 in comparison to WT values.

Not only did the CD4^+^ cells from B6.*Baff^−/−^* and B6.BTg mice display decreased and increased activation profiles, respectively, but generation of Th17 cells was also affected by BAFF. T cells isolated from the spleen, LN, or peripheral blood were stimulated with PMA + ionomycin in the presence of brefeldin A to permit accumulation of sufficient intracellular cytokines for detection by flow cytometry. In each case, percentages of CD4^+^IL-17^+^ cells were higher in B6.BTg cells and lower in B6.*Baff^−/−^* cells than in B6 WT cells (Figure 2A). CD8^+^IL-17^+^ cells were not detected in any mouse cohort (data not shown). Intracellular staining for the Th17 cell-specific transcription factor, RORγt [41], and surface staining for CCR6, a marker associated with Th17 cells [42], yielded identical patterns (Figure 2B–C). Of note, percentages of CD4^+^IL-17^+^ cells were identical in B6.BTg and B6.*Baff^−/−^*.BTg cells, indicating that membrane BAFF is dispensable for Th17 cell generation as long as soluble BAFF is plentiful.

### Direct T cell effect by BAFF in generation of Th17 cells

**Figure 3:**
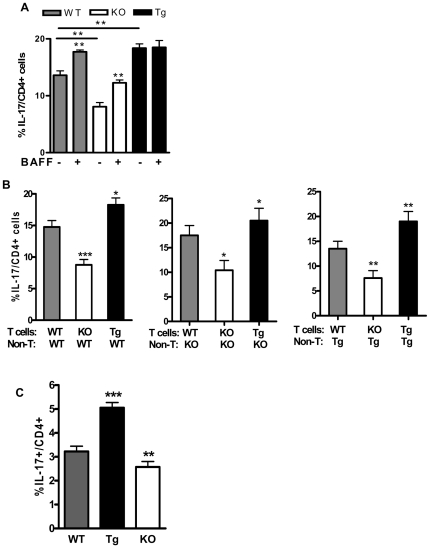
Direct effect of BAFF on T cells in generation of Th17 cells. Panel A: Spleen naive CD4^+^ cells from the indicated mice (n = 4 in each cohort) were cultured for 3 days with anti-CD3 + anti-CD28 mAb (1 µg/ml each) in the presence of irradiated syngeneic non-T cells, IL-6 (10 ng/ml), TGF-β (2 ng/ml), and anti-IL-4 + anti-IFNγ antibodies (10 µg/ml each) and in the presence or absence of exogenous BAFF (100 ng/ml). Harvested cells were stained for surface CD4 and intracellular IL-17. Significantly (p≤0.01) greater percentages of CD4^+^IL-17^+^ cells were generated in B6 WT cultures with added BAFF vs no added BAFF; in B6.*Baff^−/−^* cultures with added BAFF vs no added BAFF; and in B6.BTg cultures with no added BAFF vs WT cultures with no added BAFF. Significantly (p≤0.01) reduced percentages of CD4^+^IL-17^+^ cells were generated in B6.*Baff^−/−^* cultures with added BAFF vs B6 WT cultures with no added BAFF. Panel B: Spleen naive CD4^+^ cells from the indicated mice were co-cultured for 3 days with irradiated non-T cells from the indicated mice for 3 days as in panel A without exogenous BAFF. Harvested cells were stained for surface CD4 and intracellular IL-17. Panel C: Spleen naive CD4^+^ cells from the indicated mice (n = 2 in each cohort) were cultured for 3 days as in panel A, except that no irradiated non-T cells were added to the cultures, and plate-bound anti-CD3 mAb (2 µg/ml) was used in place of soluble anti-CD3 mAb. *, p≤0.05; **, p≤0.01; ***, p≤0.001 in comparison to WT values.

To establish a direct effect of BAFF on T cells in generating Th17 cells, we first cultured naive CD4^+^ cells from B6 WT, B6.*Baff^−/−^*, or B6.BTg mice for 3 days with anti-CD3 + anti-CD28 mAb in the presence of irradiated syngeneic non-T cells, IL-6, TGF-β, and anti-IL-4 + anti-IFNγ antibodies [43]. Exogenous BAFF (100 ng/ml) either was or was not added to these cultures. As expected, percentages of CD4^+^IL-17^+^ cells in the absence of exogenous BAFF were greatest in cultures of B6.BTg cells and were the fewest in cultures of B6.*Baff^−/−^* cells (Figure 3A). The addition of exogenous BAFF to B6 WT and B6.*Baff^−/−^* cells increased the percentages of CD4^+^IL-17^+^ cells, consistent with the increased percentages of such cells in BAFF-Tg hosts. Of note, no incremental effect of exogenous BAFF was appreciated in cultures of B6.BTg cells, suggesting that the high endogenous levels of BAFF in B6.BTg mice had already conferred a maximal BAFF-mediated signal to the T cells.

Next, co-cultures of T cells + non-T cells were established in a criss-cross fashion so that T cells from B6 WT, B6.*Baff^−/−^*, and B6.BTg mice were individually cultured with non-T cells from each of the mice. Regardless of the source of non-T cells, Th17 cell generation was the greatest in cultures containing B6.BTg T cells and was the lowest in cultures containing B6.*Baff^−/−^* T cells (Figure 3B). The source of non-T cells had little, if any, effect on the percentages of Th17 cells recovered. Moreover, differential Th17 cell generation was appreciated even in the absence of non-T cells. When naive CD4^+^ cells were stimulated with plate-bound anti-CD3 to circumvent the absolute requirement for APC in the cultures, the greatest percentages of Th17 cells again arose in B6.BTg T cell cultures, and the lowest percentages again arose in B6.*Baff^−/−^* T cell cultures (Figure 3C). Although Th17 cell generation was quantitatively greater in cultures containing irradiated APC, the differential Th17 cell response did not depend on APC.

### Promotion by BAFF of enhanced IL-6R expression and signaling in CD4^+^ cells

**Figure 4:**
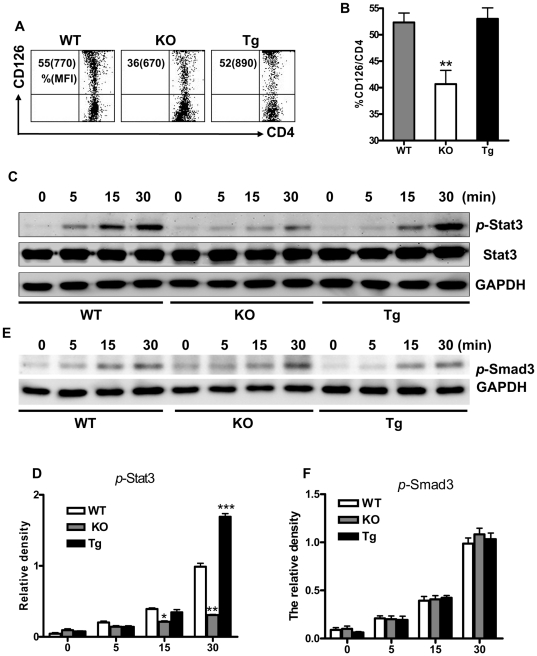
Differential CD126 expression and IL-6-triggered signaling in CD4^+^ cells from B6 WT, B6.*Baff^−/−^*, and B6.BTg mice. Panel A: LN cells were double-stained for CD4 and CD126 from the indicated mice. The dot plots reflect representative mice, with the values outside the parentheses indicating the percentages of CD126^+^ cells within the CD4^+^ population and the values inside the parentheses indicating the mean fluorescence intensity of the CD126^+^ cell population. Panel B: Results (mean ± SEM) are presented for the 3 mice in each cohort. Panels C–D: Naive CD4^+^ cells from the indicated mice were stimulated with IL-6 (10 ng/ml) + TGF-β (2 ng/ml) for the indicated periods of time (minutes) and were subjected to Western blot analysis for total Stat3, phosphorylated Stat3 (p-Stat3), and GADPH (panels C–D) and phosphorylated Smad3 (p-Smad3) and GADPH (panels E–F). The levels of p-Stat3 and p-Smad3 were normalized to GAPDH, with the ratios of p-Stat3 or p-Smad3 to GADPH at 30 minutes being arbitrarily assigned a value of 1. The results shown in panels C and E are representative of the 7 independent experiments, and the results shown in panels D and F are the mean ± SEM of these 7 experiments. *, p≤0.05; **, p≤0.01; ***, p≤0.001 in comparison to WT values.

Since IL-6 plays a central role in the generation of Th17 cells [44] and BAFF promotes IL-6 production by myeloid-lineage cells [45], [46], we viewed the IL-6 pathway as a likely participant in BAFF-driven generation of Th17 cells. Indeed, there were significantly fewer CD4^+^ cells that expressed CD126 (IL-6R α chain) in B6.*Baff^−/−^* mice than in B6 WT mice, and the intensity of CD126 staining on CD4^+^CD126^+^ cells in B6.*Baff^−/−^* mice was lower than that on the corresponding B6 WT cells (Figure 4A). Although the percentage of CD4^+^CD126^+^ cells was not greater in B6.BTg mice than in B6 WT mice, the CD126 staining intensity was much greater in the former than in the latter (Figure 4A–B). Of note, no difference in CD130 (IL-6R β chain) expression was detected among the three mouse cohorts (data not shown), suggesting that CD126 and CD130 may be differentially regulated by BAFF.

To assess the consequences of the differential expression of CD126 among B6.*Baff^−/−^*, B6.BTg, and B6 WT mice for intracellular signaling pathways, we focused on the Stat3 pathway which is vital to Th17 cell generation [47]. Naive CD4^+^ cells from B6.*Baff^−/−^*, B6.BTg, and B6 WT mice were incubated with IL-6 + TGF-β. Generation of phosphorylated (activated) Stat3 was significantly greater in B6.BTg naive CD4^+^ cells than in B6 WT naive CD4^+^ cells following 30 minutes of stimulation with IL-6 + TGF-β, whereas the reduced generation of phosphorylated Stat3 in B6.*Baff^−/−^* naive CD4^+^ cells was already apparent by 15 minutes (Figure 4C–D). Importantly, no differences in expression of TGF-β receptors I or II (data not shown) or in expression of Smad2 (data not shown) or Smad3 (Figure 4E–F) were appreciated among the three mouse cohorts.

### BAFF promotes development of EAE in association with Th17 generation

**Figure 5:**
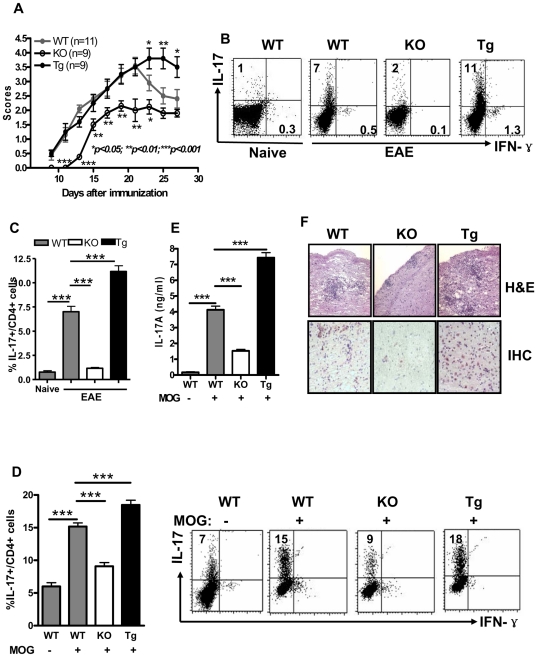
Differential severity of EAE in B6.*Baff^−/−^* and B6.BTg mice and its association with a Th17 profile. Panel A: The indicated mice were immunized with MOG_35–55_ in CFA and evaluated for clinical EAE. Results (mean ± SEM) are the aggregate of 3 independent experiments (each containing ≥3 mice per cohort). *, p<0.05; **, p<0.01; ***, p<0.001 in comparison to WT mice at the indicated time points. Panel B: T cells from the draining LN of the indicated mice were stimulated with PMA + ionomycin in the presence of brefeldin A as in Figure 2 and stained for surface CD4 or CD8 and intracellular IL-17 or IFNγ. The values in the upper left quadrants in each tracing indicate the percentages of IL-17^+^ cells (within the CD4^+^ population) in that sample, and the values in the lower right quadrants indicate the percentages of IFNγ^+^ cells (within the CD4^+^ population). Results from a representative mouse from each cohort are shown. Panel C: Results are presented for CD4^+^IL-17^+^ cells for the 3 mice in each cohort. Panel D: Draining LN cells from the MOG_35–55_ peptide-immunized mice either were (+) or were not (−) cultured with MOG_35–55_ peptide for 3 days. Percentages of CD4^+^IL-17^+^ cells were determined. Results are presented for the 6 mice in each cohort (left) or a representative mouse from each cohort (right). ***, p<0.001. Panel E: Supernatants from the cultures in panel D were assayed for IL-17A. Results are presented as mean ± SEM. Panel F: The indicated mice were sacrificed on day 25 post-immunization, and sections of their brains and spinal cords were stained with H&E and immunostained for IL-17. Results are representative of the 6 mice in each cohort. Original magnification is 400×.

To assess whether changes in BAFF expression had consequences for *in vivo* Th17 cell-mediated pathophysiology, we turned to EAE, a Th17 cell-mediated disease [37]. Following immunization with the encephalitogenic MOG_35–55_ peptide, early manifestations of EAE appeared in B6 WT and B6.BTg mice by day 7 and progressed through day 21 (Figure 5A). Although disease severity began to wane at this time in B6 WT mice, it persisted in B6.BTg mice. Strikingly, disease onset was delayed, and peak disease severity was significantly diminished, in B6.*Baff^−/−^* mice.

Not only was clinical disease a function of BAFF expression, but generation of Th17 cells was as well. In comparison to naive (non-immunized) B6 WT mice, B6 WT EAE mice harbored considerably more CD4^+^IL-17^+^ cells in their draining LN (Figure 5B–C). Importantly, this up-regulation was amplified in B6.BTg EAE mice but was largely suppressed in B6.*Baff^−/−^* EAE mice. Some up-regulation in CD4^+^IFNγ^+^ (Th1) cells in B6 WT and B6.BTg EAE mice was also noted, but these cells were substantially fewer in number than were Th17 cells (Figure 5B).

At least part of the up-regulated Th17 cell response was specific for the MOG_35–55_ peptide used to induce EAE. Draining LN cells were harvested on day 16 post-immunization (when clinical disease was already substantial and continuing to increase in severity) and were stimulated in culture with MOG_35–55_ peptide for 3 days. In comparison to unstimulated B6 WT cultures, B6 WT cultures stimulated with MOG_35–55_ peptide harbored increased percentages of CD4^+^IL-17^+^ cells and increased concentrations of IL-17A (Figure 5D–E). Each of these levels was further increased in MOG_35–55_ peptide-stimulated B6.BTg cultures but was substantially attenuated in corresponding B6.*Baff^−/−^* cultures.

Of great importance, pathological evaluation of the CNS paralleled the clinical findings, with inflammation being the greatest in B6.BTg mice and being the mildest in B6.*Baff^−/−^* mice (Figure 5F). Immunostaining documented infiltration of Th17 cells into the CNS, with the greatest number of Th17 cells detected in B6.BTg mice and the fewest number of Th17 cells detected in B6.*Baff^−/−^* mice. Although the Ag-specificity and pathogenic potency of these infiltrating Th17 cells remain speculative, our observations are consistent with an important contributory role for BAFF in the development of disease-promoting Th17 cells.

## Discussion

Given its prominent role as a B cell survival factor [12]–[14], it is not surprising that BAFF has received considerable attention as a candidate therapeutic target in disorders associated with B cell hyperactivity. The clinically meaningful efficacy of the anti-BAFF mAb, belimumab, in SLE clinical trials [48], [49] provides *prima facie* evidence that BAFF antagonism can be beneficial in at least some autoimmune disorders.

Neutralization (elimination) of BAFF may also be beneficial in T cell-mediated disorders, inasmuch as T cells express the BAFF receptor BR3 [35]. Accordingly, we studied mice at both ends of the “BAFF spectrum”; that is, mice that were completely devoid of BAFF (B6.*Baff^−/−^* mice) and mice that constitutively over-expressed BAFF (B6.BTg mice).

As expected, the percentages of B cells were decreased in B6.*Baff^−/−^* mice and increased in B6.BTg mice, with total T cell numbers, CD4^+^ cell numbers, and, to a lesser extent, CD8^+^ cell numbers each being decreased in B6.*Baff^−/−^* mice and increased in B6.BTg mice (Figure 1A–B). Moreover, CD4^+^ cells in B6.BTg mice displayed a more “activated” phenotype, whereas CD4^+^ cells in B6.*Baff^−/−^* mice displayed a less “activated” phenotype (Figure 1C).

The *in vivo* effect of BAFF extended to Th17 cells, with multiple markers of Th17 cells being increased in B6.BTg mice and decreased in B6.*Baff^−/−^* mice (Figure 2). Thus, not only does BAFF affect Th1 and Th2 cell responses [36], but it also affects Th17 cell responses. Of note, B6.BTg mice (which express membrane BAFF and over-express soluble BAFF) and B6.*Baff^−/−^*. BTg mice (which over-express soluble BAFF but have no membrane BAFF) harbored similar percentages of Th17 cells, indicating that membrane BAFF is dispensable for Th17 cell generation as long as soluble BAFF is plentiful. Indeed, addition of exogenous soluble BAFF to cultures of naive CD4^+^ cells from B6 WT or B6.*Baff^−/−^* mice augmented generation of Th17 cells (Figure 3A). Whether membrane BAFF in the absence of soluble BAFF can promote Th17 cell generation (as might occur in patients treated with an agent specific for soluble BAFF) remains to be established.

Since differences in levels of BAFF expression have profound consequences for B cells, effects of BAFF on Th17 cells could, in principle, be secondary to alterations in B cell numbers and/or phenotype. Nonetheless, at least part of BAFF's ability to promote Th17 cells lies in its direct effect on T cells. Cultures containing naive CD4^+^ cells from B6.BTg mice always generated the greatest number of Th17 cells regardless of the source of irradiated APC (including APC from B6.*Baff^−/−^* mice), and cultures containing naive CD4^+^ cells from B6.*Baff^−/−^* mice always generated the fewest number of Th17 cells (even when the irradiated APC came from B6.BTg mice) (Figure 3B). Indeed, differential Th17 cell responses were also observed in cultures devoid of any non-T cells (Figure 3C). Future studies will be needed to determine whether this non-T cell-independent effect of BAFF on *in vitro* Th17 cell generation is replicated *in vivo*.

Since blockade of the IL-6 pathway can suppress Th17 cell differentiation [50], the differential expression of CD126 on CD4^+^ cells among B6 WT, B6.*Baff^−/−^*, and B6.BTg mice and the resulting differential responsiveness to IL-6 (Figure 4A–D) suggest a prominent role for the IL-6 pathway in BAFF-driven Th17 cell generation. Of note, the absence of differential Smad activation (Figure 4E–F) suggests that the TGF-β pathway plays little, if any, role in this BAFF-driven process. Regardless, the interlocking of the IL-6 and BAFF pathways may have therapeutic ramifications, since an IL-6 antagonist (tocilizumab) is already approved for the treatment of one autoimmune inflammatory disorder (rheumatoid arthritis) and, in principle, could be beneficial in others as well. Additional studies will be needed to fully delineate the molecular underpinnings that connect the BAFF and IL-6 pathways pathophysiologically and therapeutically.

Although the clinical efficacy of BAFF antagonism has, to date, been demonstrated only in SLE [48], [49], clinical efficacy could also emerge in several other autoimmune diseases, including MS. Indeed, BAFF expression is increased in the monocytes and T cells in some MS patient subsets [51], and it is markedly up-regulated in the CNS demyelinating plaques [52]. That is, BAFF expression is often increased both in those immune cells that generate and effect the pathologic autoimmune response in MS as well as at the sites of tissue pathology and damage.

In B6.*Baff^−/−^* mice, development of EAE is delayed and less severe than in B6 WT mice. Conversely, the duration of EAE in B6.BTg mice is prolonged (Figure 5A). These observations dovetail well with previous reports of the associations between up-regulation of BAFF in the CNS with development of EAE [53] and successful treatment with glatiramer acetate with the down-regulation of BAFF expression in the CNS [54].

Importantly, generation of Th17 cells paralleled the clinical disease in our mice, with B6.BTg EAE mice harboring considerably more, and B6.*Baff^−/−^* EAE mice harboring considerably fewer, CD4^+^IL-17^+^ cells in their draining LN than B6 WT EAE mice (Figure 5B–C). These CD4^+^IL-17^+^ cells likely included MOG_35–55_ peptide-specific (encephalitogenic) Th17 cells (Figure 5D–E). Of note, the up-regulation in Th17 cells in both B6.BTg and B6 WT mice was much greater than the corresponding up-regulation in Th1 cells (Figure 5B), suggesting that Th17 cells may be playing a more important role in EAE than do Th1 cells. Although the contributions of IL-17A and IL-17F themselves to the neuro-inflammatory process may be limited [55], Th17 cells have been shown to directly interact with neuronal cells in demyelinating lesions and adversely affect neuronal function [56].

In any case, CNS pathology and infiltration of Th17 cells into the CNS paralleled the clinical findings, with each being the greatest in B6.BTg mice and being the mildest in B6.*Baff^−/−^* mice (Figure 5F). Whether these Th17 cells represent cells that had differentiated in the periphery and then migrated to the CNS or whether these Th17 cells represent undifferentiated T cells that infiltrated the CNS and underwent differentiation to the Th17 lineage *in situ* remains to be established.

Others have previously reported delayed onset and reduced severity of disease in mice treated prior to induction of EAE with a fusion protein that neutralizes both BAFF and APRIL [57]. Our findings extend these observations in that we unequivocally document that neutralization (elimination) of BAFF alone (without affecting APRIL) leads to delayed onset and reduced disease severity. It is unlikely that these salutary effects are secondary consequences of B cell depletion, since development of EAE in B cell-deficient mice is at least as rapid and severe as in B cell-sufficient mice [58]. However, both in the study that pharmacologically neutralized BAFF and in our study that genetically manipulated BAFF expression, the degree of benefit actually derived from inhibition of (pathogenic) Th17 cell responses could not be quantified, since (pathogenic) autoantibody responses were likely also inhibited.

Nonetheless, experience in CIA, another disease largely driven by Th17 cells [59], is telling. When local BAFF expression in the joints of mice with CIA was silenced, not only was disease ameliorated but Th17 cell generation was diminished as well. Conversely, when local BAFF expression in these joints was augmented, disease intensity and Th17 cell generation were augmented in parallel [60]. Importantly, the ability of exogenous BAFF to aggravate CIA was abrogated in the few IL-17-deficient hosts who did develop disease, indicating that Th17 cells play an important role in BAFF-aggravated CIA and, by extension, may play an important role in other Th17 cell-dependent autoimmune diseases.

In summary, our observations document a direct effect of BAFF on T cells in promoting Th17 cells. These BAFF-driven Th17 cell responses contribute to the clinical and pathological severity of EAE. Given the contribution of BAFF-driven Th17 cell responses to another Th17 cell-dependent autoimmune disease (CIA), BAFF should be viewed as a favorable candidate therapeutic target in Th17 cell-dependent autoimmune diseases.
